# A risk prediction model for gastrointestinal bleeding in coronary heart disease patients undergoing dual antiplatelet therapy after percutaneous coronary intervention

**DOI:** 10.12669/pjms.41.12.13169

**Published:** 2025-12

**Authors:** Yuncong Ma, Suxia Fang

**Affiliations:** 1 Yuncong Ma, Department of Cardiology, Zhejiang University School of Medicine Second Affiliated Hospital Linping Campus, Hangzhou, Zhejiang Province 311100, P.R. China; 2 Suxia Fang, Department of Cardiology, Zhejiang University School of Medicine Second Affiliated Hospital Linping Campus, Hangzhou, Zhejiang Province 311100, P.R. China

**Keywords:** Coronary heart disease, PCI, Dual antiplatelet therapy, Gastrointestinal bleeding, Risk prediction mode

## Abstract

**Objective:**

To develop a risk prediction model for gastrointestinal bleeding in coronary heart disease (CHD) patients who undergo dual antiplatelet therapy (DAPT) after percutaneous coronary intervention (PCI).

**Methodology:**

The clinical data of 185 CHD patients who received DAPT after PCI and were admitted to the First People’s Hospital of Linping District from November 2023 to March 2025 were retrospectively selected. Among them, 29 patients with gastrointestinal bleeding comprised the bleeding group, and 156 patients without gastrointestinal bleeding comprised the non-bleeding group. Logistic model analysis was used to identify independent risk factors for gastrointestinal bleeding during DAPT after PCI, and a prediction model was constructed. The receiver operating characteristic (ROC) curve of the prediction model for gastrointestinal bleeding, along with the area under the curve (AUC), was calculated to evaluate the predictive value of the model.

**Results:**

Logistic model analysis identified age, chronic renal insufficiency, history of digestive tract disease and infarction, PCI procedure duration, creatinine clearance rate, and use of gastric mucosal protective agents as risk factors of gastrointestinal bleeding (P < 0.05). AUCs of the constructed prediction model for age, chronic renal insufficiency, history of digestive tract disease, history of myocardial infarction, PCI procedure duration, creatinine clearance rate, and gastric mucosal protective agent were 0.690 (95% confidence interval (CI) 0.594 - 0.778), 0.714 (0.616 - 0.807), 0.728 (0.633 - 802), 0.742 (0.654 - 0.828), 0.837 (0.763 - 0.901), 0.783 (0.691 - 0.870), and 0.754 (0.663 - 0.838), respectively.

**Conclusion:**

The risk factors for gastrointestinal bleeding in CHD patients during DAPT after PCI include advanced age, chronic renal insufficiency, digestive tract disease history, myocardial infarction history, PCI procedure duration, creatinine clearance rate, and non-application of gastric mucosal protective agents. The ROC curve drawn based on the above factors can effectively predict gastrointestinal bleeding during DAPT. This model can be used to develop clinically targeted interventions aimed at preventing gastrointestinal bleeding and improving patients’ prognosis.

## INTRODUCTION

Percutaneous coronary intervention (PCI), with its advantages of minimal invasiveness and high efficacy, has become a core technology for improving myocardial blood supply in patients with coronary heart disease (CHD) and reducing the risk of acute cardiovascular events.^1^-^3^ However, the procedure is associated with severe complications, such as in-stent thrombosis.^4^ Dual antiplatelet therapy (DAPT), which was shown to significantly reduce the incidence of in-stent thrombosis and major adverse cardiovascular events (MACE) by inhibiting platelet aggregation, has now become the standard treatment regimen after PCI.^5^-^7^

Although DAPT plays an irreplaceable role in cardiovascular protection, antiplatelet drugs reduce prostaglandin synthesis by inhibiting cyclooxygenase-1 (COX-1), thereby impairing the gastric mucosal protective barrier and interfering with platelet hemostatic function, which leads to a significantly increased risk of gastrointestinal bleeding.^8^,^9^ Indeed, clinical data show that the incidence of gastrointestinal bleeding in patients receiving DAPT after PCI can reach 3% to 8%.^10^ This side effect often merits clinical interruption or adjustment of antiplatelet therapy, which in turn increases the risk of adverse cardiovascular events, forming a vicious cycle of “bleeding - drug withdrawal - thrombosis” that seriously affects patient prognosis and long-term quality of life.^11^-^13^

In current clinical practice, the assessment of gastrointestinal bleeding risk during DAPT primarily relies on the clinician’s empirical judgment or simple scoring tools, lacking specificity for the population receiving DAPT after PCI for CHD. Therefore, a precise risk prediction model is needed for this specific population to achieve individualized stratification of bleeding risk. In recent years, risk prediction models based on methods such as multivariate regression and machine learning have gained widespread use in the cardiovascular field.^14^-^16^ However, while existing models such as PRECISE-DAPT and HAS-BLED are widely applied for bleeding risk prediction in a broad patient population, they are not specifically tailored for patients undergoing percutaneous coronary intervention (PCI) while receiving dual antiplatelet therapy (DAPT).^17^,^18^ These models primarily focus on general cardiovascular risk factors and fail to address the unique clinical characteristics of PCI patients, such as chronic renal insufficiency, a history of gastrointestinal diseases, and the duration of the PCI procedure. Therefore, a model that specifically targets the post-PCI patient population undergoing DAPT, incorporating clinically significant factors, will have superior predictive accuracy for this high-risk group and will provide more precise, individualized, and clinically actionable predictions compared to existing models.

This retrospective cohort study aimed to identify independent risk factors for gastrointestinal bleeding in patients with CHD receiving DAPT after PCI, and to construct and validate a precise risk prediction model that could be used to design individualized preventive strategies and verify their effectiveness through clinical trials.

## METHODOLOGY

Clinical data of 185 patients with coronary heart disease who received DAPT after PCI and were admitted to the First People’s Hospital of Linping District from November 2023 to March 2025 were retrospectively selected. Among them, 29 patients with gastrointestinal bleeding were assigned to the bleeding group, and 156 patients without gastrointestinal bleeding were assigned to the non-bleeding group. Gastrointestinal bleeding was confirmed by symptoms such as hematemesis, melena, or hematochezia, combined with a positive fecal occult blood test, and bleeding lesions identified by gastroscopy/colonoscopy, or a decrease in hemoglobin of≥ 20 g/L from baseline, requiring transfusion of ≥ 2 units (excluding other sources of bleeding).

### Ethical Approval:

The study was approved by the Ethics Committee of the First People’s Hospital of Linping District (Approval number: 2023059), dated January 23, 2021.

### Inclusion criteria:

- Meeting the diagnostic criteria for coronary heart disease.^19^
- Underwent successful PCI (coronary artery stenosis ≥ 50% with complete surgical records).
- Regularly receiving post-operative DAPT (aspirin + P2Y12 antagonist).
- Complete clinical data (including baseline characteristics, examination results, and treatment records).
- Expected survival time ≥ 6 months.
- No history of active gastrointestinal bleeding before PCI.

### Exclusion criteria:

- Patients in the acute phase of ST-segment elevation myocardial infarction.
- Severe liver and kidney dysfunction (Child-Pugh class C or eGFR < 30 ml/min).
- Congenital or acquired coagulation disorders.
- History of malignant tumors or current malignant tumors.
- Long-term combined use of anticoagulants (warfarin, new oral anticoagulants, etc.).
- History of previous in-stent thrombosis.
- Missing follow-up data or high risk of loss to follow-up (e.g., cognitive impairment, inability to cooperate with follow-up).

### Observation Indicators:

The following data were collected:

- General data, including age, gender, time from onset to admission, drinking history, smoking history, underlying diseases (diabetes, hypertension), presence of chronic renal insufficiency, presence of digestive tract disease history, presence of myocardial infarction history;
- Clinical information, such as number of diseased vessels, lesion location, degree of stenosis, PCI duration;
- Biochemical indicators, such as platelet count, hemoglobin, creatinine clearance rate,
- The use of gastric mucosal protective agents.

### Statistical Analysis:

Data were processed using SPSS 26.0 software. The count data were described as cases and analyzed using the χ^2^ test. Measurement data were tested for homogeneity of variance (Bartlett test) and normality (Kolmogorov-Smirnov test). Data with homogeneous variance and an approximate normal distribution were described as mean ± standard deviation and analyzed using the t-test. Influencing factors were analyzed using multiple linear regression, and the results were presented as odds ratios (OR) with the 95% confidence intervals (CI). A receiver operating characteristic (ROC) curve of the prediction model for gastrointestinal bleeding during DAPT after PCI was constructed, and the area under the curve (AUC) was calculated to evaluate its predictive value. The significance level was set at α = 0.05.

## RESULTS

There were no statistically significant differences between the two groups in terms of gender, drinking history, smoking history, underlying diseases (diabetes, hypertension), time from onset to admission, number of diseased vessels, lesion location, degree of stenosis, platelet count, or hemoglobin level (P > 0.05). However, as shown in Table-I, statistically significant differences were observed in age, presence or absence of chronic renal insufficiency, presence or absence of digestive tract disease history, presence or absence of myocardial infarction history, PCI procedure duration, creatinine clearance rate, and use of gastric mucosal protective agents (P < 0.05).

**Table-I:** Comparison of General Data between the Two Groups.

Items		Bleeding Group(n=29)	Non-bleeding Group(n=156)	t/χ^2^ Value	P Value
Gender [n(%)]	Male	12(41.38)	82(52.56)	1.224	0.269
	Female	17(58.62)	74(47.44)		
Drinking History [n(%)]	Yes	11(37.93)	38(24.36)	2.313	0.128
	No	18(62.07)	118(75.64)		
Smoking History [n(%)]	Yes	16(55.17)	73(46.79)	0.688	0.407
	No	13(44.83)	83(53.21)		
With or without Diabetes [n(%)]	Yes	12(41.38)	42(26.92)	2.473	0.116
	No	17(58.62)	114(73.08)		
With or without Hypertension [n(%)]	Yes	14(48.82)	49(31.41)	3.097	0.078
	No	15(51.72)	107(68.59)		
Time from Onset to Admission(*Χ̅±S*, h)		3.41±0.62	3.23±0.71	1.277	0.203
Number of Diseased Vessels [n(%)]	Single vessel	18(62.07)	107(68.59)	0.475	0.491
	Multiple vessels	11(37.93)	49(31.41)		
Lesion Location [n(%)]	Right coronary artery	11(37.93)	61(39.10)	0.356	0.837
	Circumflex artery	7(24.14)	44(28.21)		
	Anterior descending artery	11(37.93)	51(32.69)		
Degree of Stenosis [n(%)]	Mild	10(34.48)	49(31.41)	0.108	0.947
	Moderate	12(41.38)	67(42.95)		
	Severe	7(24.14)	40(25.64)		
Platelet Count(*Χ̅±S*, ×10^9^/L)		209.86±28.66	212.04±20.15	0.488	0.626
Hemoglobin(*Χ̅±S*, g/L)		123.37±21.51	122.69±21.71	0.155	0.877
Age [n(%)]	≤60 years old	10(34.48)	113(72.44)	15.809	0.000
	>60 years old	19(65.52)	43(27.56)
Chronic Renal Insufficiency [n(%)]	Yes	18(62.07)	30(19.23)	23.356	0.000
	No	11(37.93)	126(80.77)
History of Digestive Tract Disease [n(%)]	Yes	19(65.52)	31(19.87)	25.834	0.000
	No	10(34.48)	125(80.13)		
History of Myocardial Infarction [n(%)]	Yes	20(68.97)	32(20.51)	28.410	0.000
	No	9(31.03)	124(79.49)		
PCI procedure duration (*Χ̅±S*, min)		98.06±15.03	80.51±11.38	7.226	0.000
Creatinine Clearance Rate (*Χ̅±S*, ml/min)		58.80±18.12	79.11±20.22	5.044	0.000
Gastric Mucosal Protective Agent [n(%)]	Applied	7(24.14)	117(75.00)	28.624	0.000
	Not Applied	22(75.86)	39(25.00)		

Logistic model analysis with gastrointestinal bleeding as the dependent variable, and the identified indicators with statistically significant differences as independent variables (Table-II) for assignment) confirmed that age, chronic renal insufficiency, history of digestive tract disease, history of myocardial infarction, PCI procedure duration, creatinine clearance rate, and non-application of gastric mucosal protective agents are all independent risk factors for gastrointestinal bleeding in patients with coronary heart disease during DAPT after PCI (P < 0.05; Table-III).

**Table-II:** Assignment.

Variable	Assignment
Age	≤ 60 years old = 0, > 60 years old = 1
Chronic Renal Insufficiency	No = 0, Yes = 1
History of Digestive Tract Disease	No = 0, Yes = 1
History of Myocardial Infarction	No = 0, Yes = 1
PCI procedure duration	Original value
Creatinine Clearance Rate	Original value
Gastric Mucosal Protective Agent	Not applied = 0, Applied = 1

**Table-III:** Analysis of Risk Factors for Gastrointestinal Bleeding in Patients with Coronary Heart Disease During DAPT After PCI.

Variable	B Value	S.E. Value	Waldχ^2^ Value	P Value	OR Value	95%CI
Age	1.208	0.382	10.006	<0.001	3.348	1.569~7.144
Chronic Renal Insufficiency	1.697	0.611	7.718	<0.001	5.460	3.158~9.440
History of Digestive Tract Disease	1.416	0.496	8.146	<0.001	4.119	2.770~6.125
History of Myocardial Infarction	1.543	0.505	9.335	<0.001	4.678	2.853~7.671
PCI procedure duration	1.526	0.623	6.002	<0.001	4.601	3.340~6.339
Creatinine Clearance Rate	1.685	0.599	7.915	<0.001	5.394	4.248~6.848
Gastric Mucosal Protective Agent	1.398	0.437	10.240	<0.001	4.049	2.760~5.939

**Fig.1:**
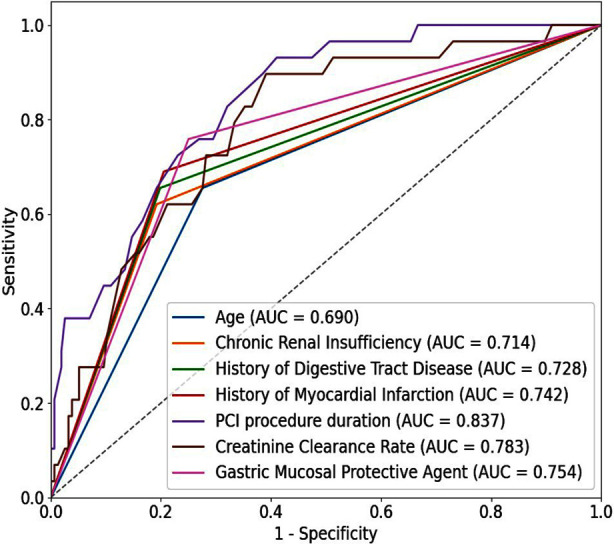
ROC Curve of the Prediction Model for Gastrointestinal Bleeding in Patients with Coronary Heart Disease During DAPT After PCI.

The ROC curve of the generated prediction model was plotted using SPSS. As shown in Fig.1, the AUCs of age, chronic renal insufficiency, history of digestive tract disease, history of myocardial infarction, PCI procedure duration, creatinine clearance rate, and gastric mucosal protective agent were 0.690 (95% CI 0.594 - 0.778), 0.714 (0.616 - 0.807), 0.728 (0.633 - 802), 0.742 (0.654 - 0.828), 0.837 (0.763 - 0.901), 0.783 (0.691 - 0.870), and 0.754 (0.663 - 0.838), respectively (Table-IV).

**Table-IV:** ROC Curve Analysis of the Predictive Model for Gastrointestinal Bleeding During DAPT in Patients with Coronary Heart Disease After PCI.

Variable	AUC	S.E. Value	P Value	95%CI
Age	0.690	0.048	<0.001	0.584~0.778
Chronic Renal Insufficiency	0.714	0.048	<0.001	0.616~0.807
History of Digestive Tract Disease	0.728	0.048	<0.001	0.633~0.820
History of Myocardial Infarction	0.742	0.044	<0.001	0.654~0.828
PCI procedure duration	0.837	0.035	<0.001	0.763~0.901
Creatinine Clearance Rate	0.783	0.045	<0.001	0.691~0.870
Gastric Mucosal Protective Agent	0.754	0.044	<0.001	0.663~0.838

## DISCUSSION

This study identified age, chronic renal insufficiency, history of digestive tract diseases, history of myocardial infarction, PCI procedure duration, creatinine clearance rate, and application of gastric mucosal protective agents as independent risk factors for gastrointestinal bleeding during DAPT.

DAPT-related gastrointestinal bleeding after PCI treatment in patients with CHD has always been an important issue affecting the safety of the treatment.^20^ Gastrointestinal bleeding may lead to an increased risk of thrombosis due to forced interruption of antiplatelet therapy, seriously impacting the prognosis.^21^ Therefore, identifying the risk factors for gastrointestinal bleeding during DAPT and establishing effective prediction tools are of great significance for clinical risk stratification and individualized intervention.

The identified risk factors of gastrointestinal bleeding during DAPT after PCI in coronary heart disease patients in this study are consistent with previous reports. The results further confirm that the occurrence of gastrointestinal bleeding is the result of the combined action of multiple factors.^22^,^23^ Age negatively affects the ability of the gastrointestinal mucosa to repair and increases vascular fragility.^24^ Additionally, elderly patients often have numerous underlying diseases and reduced tolerance to drugs, which makes the direct stimulation of the gastric mucosa by antiplatelet drugs and the inhibitory effect on prostaglandin synthesis more significant, thereby increasing the risk of bleeding.^25^

Patients with chronic renal insufficiency are prone to gastrointestinal mucosal damage due to metabolic disorders, accumulation of uremic toxins, and abnormal platelet function.^26^ As a core indicator of decreased renal function, reduced creatinine clearance directly reflects the decline in renal excretion function, which not only leads to the accumulation of antiplatelet drugs in the body and aggravates mucosal damage, but may also further increase the risk of bleeding by affecting the synthesis of coagulation factors and platelet function.^27^ Therefore, reduced creatinine clearance and chronic renal insufficiency synergistically constitute significant risk factors.^28^,^29^ A history of digestive tract diseases (such as gastric ulcer, gastritis) is a clear basis for gastrointestinal bleeding, as DAPT drugs (aspirin, clopidogrel, etc.) can aggravate existing lesions or induce bleeding by directly stimulating the mucosa or affecting the repair mechanism. In this study, creatinine clearance yielded the highest area under the curve (AUC = 0.943) among all predictors, indicating that renal function is a powerful determinant of gastrointestinal bleeding risk. Multiple pathophysiological pathways support this finding.^30^-^32^ Impaired renal function alters the pharmacokinetics of antiplatelet agents by reducing their renal clearance, which may lead to drug accumulation and increased mucosal toxicity.^33^ Additionally, chronic kidney disease (CKD) is associated with platelet dysfunctions, such as impaired adhesion, aggregation, and granule secretion, further predisposing patients to bleeding complications during DAPT.^34^ Finally, uremia-induced alterations in coagulation pathways contribute to an imbalance between procoagulant and anticoagulant factors, including reduced synthesis of coagulation factors and enhanced fibrinolysis.^35^ These overlapping mechanisms synergistically elevate bleeding susceptibility in CKD patients, which may explain the strong predictive value observed in the described model.

Clinical studies have confirmed a significantly increased risk of DAPT-related bleeding in people with a history of digestive tract diseases.^36^ Patients with a history of myocardial infarction usually have more complex vascular lesions or long-term drug exposure, and the stress response during acute myocardial infarction may cause ischemia of the gastrointestinal mucosa, further increasing the risk of bleeding during DAPT.^37^ Prolonged PCI procedures often indicate greater lesion complexity that requires more extensive procedural manipulation. These prolonged interventions are associated with several physiological mechanisms related to bleeding that may collectively explain the strong association between PCI procedure duration and bleeding risk observed in the prediction model.^38^-^41^ Prolonged exposure to anticoagulants, such as unfractionated heparin, during the surgery increases the risk of systemic coagulation disturbances.^41^ Furthermore, an extended procedure requires repeated use or a high volume of contrast agent, which may damage the vascular endothelium and impair mucosal defense. Intraoperative hemodynamic instability and sympathetic activation may cause transient gastrointestinal mucosal ischemia and hypoperfusion, leading to mucosal injury and impaired healing. Furthermore, prolonged operation time correlates with increased oxidative stress and systemic inflammation, which can further disrupt gastrointestinal mucosal integrity and coagulation balance.^38^-^41^

Absence of gastric mucosal protective therapy directly weakens the defense of the gastrointestinal mucosa. According to the 2020 ESC Guidelines for non-ST elevation acute coronary syndromes and the 2023 ACC/AHA updates, prophylactic PPI therapy is recommended in patients at high risk of gastrointestinal bleeding who are undergoing DAPT.^42^,^43^ High-risk features include prior history of peptic ulcer disease, age over 65 years, concomitant use of NSAIDs or anticoagulants, and Helicobacter pylori infection. Commonly used PPIs include omeprazole (20 mg/day), esomeprazole (20–40 mg/day), and pantoprazole (40 mg/day). These agents are effective in reducing mucosal damage through gastric acid suppression and have demonstrated protective benefit in several randomized controlled trials. Routine PPI co-administration at the initiation of DAPT should be strongly considered in high-risk patients and tailored according to individual bleeding risk profiles. In this study, the proportion of patients in the bleeding group who did not use gastric mucosal protective agents was higher, which highlights the importance of standardized combined use of protective agents.^44^

The ROC curve of the prediction model, generated based on the identified risk factors, showed good predictive performance, suggesting that the model can effectively identify high-risk patients and provide a practical tool for clinical intervention. The advantage of this model is that it integrates clinically easily accessible indicators, does not require complex testing, and is convenient for promotion and application in hospitals at all levels. The model will allow clinicians to implement individualized antithrombotic strategies based on bleeding risk stratification. According to current clinical guidelines (ESC 2020; ACC/AHA 2023), patients at high bleeding risk may benefit from shortened DAPT duration (e.g., 1–3 months), early transition to P2Y12 inhibitor monotherapy, and the careful selection of antiplatelet agents with lower bleeding risk profiles, such as clopidogrel. Additionally, routine co-administration of proton pump inhibitors (PPIs) is recommended for patients with a history of gastrointestinal disorders or other bleeding risk factors. These strategies aim to balance bleeding prevention with ischemic protection, and the proposed model may serve as a valuable tool to guide such decision-making in clinical practice. For instance, high-risk patients identified using the model would benefit from the combined use of gastric mucosal protective agents, such as PPIs, along with dynamic follow-up and continuous monitoring of gastrointestinal symptoms and renal function. For patients with reduced creatinine clearance, drug doses should be adjusted promptly to prevent cumulative damage. Patients with a history of digestive tract diseases can be screened and treated for underlying conditions, such as eradicating Helicobacter pylori, before starting DAPT to reduce potential bleeding risks. Additionally, patient education should focus on guiding patients to recognize early symptoms of bleeding and seek medical treatment promptly, thereby improving compliance.

Differentiated follow-up plans should be formulated according to varying risk levels, with high-risk patients receiving increased follow-up frequency supplemented by targeted diet and lifestyle interventions. The medical system can use information tools to embed risk assessment into electronic medical records, automatically trigger intervention suggestions, and promote multi-disciplinary collaboration between cardiology and gastroenterology departments to jointly formulate personalized plans. This approach will minimize the risk of bleeding while ensuring the anti-ischemic effect of the therapy and ultimately improving treatment safety and patient prognosis.

### Strength of the Study:

This study presents several key strengths. First, it specifically targets high-risk patients undergoing PCI who are receiving DAPT— a population often underrepresented in existing bleeding risk prediction models. By focusing on this clinically important cohort, the model addresses a crucial gap in current literature. Second, it incorporates easily accessible and clinically relevant variables, including creatinine clearance, a history of gastrointestinal disease, and a previous myocardial infarction, thereby enhancing both its practicality and applicability. Third, the model demonstrated robust predictive performance, with creatinine clearance alone yielding an AUC of 0.943, indicating its strong potential for clinical implementation. To enhance the model’s robustness and generalizability, future research should focus on external validation using prospective, multicenter cohorts. Additionally, integration of machine learning algorithms—such as random forests, XGBoost, or neural networks—may further improve performance by capturing complex, nonlinear interactions among variables that traditional logistic regression may overlook. The real-world impact of the model should be evaluated through prospective interventional studies. By embedding the model into electronic health record (EHR) systems, future studies may enable clinicians to receive real-time risk alerts and decision support. This could facilitate individualized preventive strategies, such as the early initiation of proton pump inhibitors, adjustments to antiplatelet therapy, or closer clinical monitoring. Ultimately, such integration could help establish a closed-loop risk management pathway that translates predictive insights into actionable clinical interventions.

### Limitations:

This study has several limitations that warrant consideration. First, the relatively small sample size (185 patients, including only 29 bleeding events) limits the statistical power and may compromise the robustness and generalizability of the model, particularly in predicting rare outcomes such as gastrointestinal bleeding. The retrospective and single-center nature of the study further limits its external validity, as patient characteristics may not accurately reflect those of broader clinical populations. Second, a significant class imbalance exists between the bleeding and non-bleeding groups (29 vs. 156), which may have biased the model towards non-bleeding predictions, thereby increasing the false-negative rate. At the time of analysis, resampling techniques such as SMOTE or bootstrapping were not applied, and the weighted logistic regression was not implemented to address this imbalance. This may have reduced the model’s discriminatory capacity for rare events. Third, several critical clinical factors known to influence the risk of gastrointestinal bleeding were not included due to limitations in the availability of retrospective data. These include the type and dosage of antiplatelet agents (e.g., clopidogrel vs. ticagrelor), concomitant use of NSAIDs or anticoagulants, Helicobacter pylori infection status, and adherence to medication. The omission of these variables may have introduced residual confounding and reduced model accuracy. Fourth, the model has not undergone internal validation (e.g., k-fold cross-validation or bootstrapping) or external validation using independent datasets. Without such procedures, the risk of overfitting cannot be excluded, and the stability and generalizability of the model remain uncertain. Future prospective multicenter studies are needed to validate and refine the model. Lastly, this study only assessed bleeding events during hospitalization or shortly after PCI. Long-term follow-up data (>6–12 months) were unavailable, limiting our ability to evaluate the model’s predictive performance over time. Late-onset bleeding events may be missed, and time-dependent risk factors may remain unidentified. Future studies should incorporate extended follow-up to capture evolving bleeding risks and validate long-term clinical utility.

## CONCLUSION

The risk factors for gastrointestinal bleeding in post-PCI CHD patients undergoing DAPT are complex and diverse, and include age, chronic renal insufficiency, history of digestive tract diseases, history of myocardial infarction, PCI procedure duration, creatinine clearance rate, and the use of gastric mucosal protective therapy. The developed prediction model, which incorporates these independent risk factors, demonstrated good performance and may be used for risk stratification. Targeted interventions, such as the standardized application of gastric mucosal protective agents, optimization of drug regimens, and strengthening of monitoring, should be adopted to reduce the incidence of bleeding, ensure treatment safety, and improve patient prognosis.

### Authors’ contributions:

**YM:** Study design, literature search and manuscript writing.

**YM and SF** were involved in data collection, data analysis, interpretation. Critical Review.

**YM:** Manuscript revision and validation and is responsible for the integrity of the study.

All authors have read and approved the final manuscript.

## References

[1] Hasbani NR, Ligthart S, Brown MR, Heath AS, Bebo A, Ashley KE (2022). American Heart Association's Life's Simple 7: Lifestyle Recommendations, Polygenic Risk, and Lifetime Risk of Coronary Heart Disease. Circulation.

[2] Ren XL, Liu Y, Chu WJ, Li ZW, Zhang SS, Zhou ZL (2023). Blood levels of omega-6 fatty acids and coronary heart disease: a systematic review and metaanalysis of observational epidemiology. Crit Rev Food Sci Nutr.

[3] Akbari T, Al-Lamee R (2022). Percutaneous Coronary Intervention in Multi-Vessel Disease. Cardiovasc Revascularization Med Mol Interv.

[4] Modi K, Soos MP, Mahajan K (2025). Stent Thrombosis. StatPearls. StatPearls Publishing.

[5] Rashedi S, Keykhaei M, Sato A, Steg PG, Piazza G, Eikelboom JW (2025). Anticoagulation and Antiplatelet Therapy for Atrial Fibrillation and Stable Coronary Disease: Meta-Analysis of Randomized Trials. J Am Coll Cardiol.

[6] Capodanno D, Mehran R, Krucoff MW, Baber U, Bhatt DL, Capranzano P (2023). Defining Strategies of Modulation of Antiplatelet Therapy in Patients with Coronary Artery Disease: A Consensus Document from the Academic Research Consortium. Circulation.

[7] Gragnano F, Cao D, Pirondini L, Franzone A, Kim HS, Von Scheidt M (2023). P2Y12 Inhibitor or Aspirin Monotherapy for Secondary Prevention of Coronary Events. J Am Coll Cardiol.

[8] Matsumura-Nakano Y, Shizuta S, Komasa A, Morimoto T, Masuda H, Shiomi H (2019). Open-Label Randomized Trial Comparing Oral Anticoagulation with and Without Single Antiplatelet Therapy in Patients with Atrial Fibrillation and Stable Coronary Artery Disease Beyond 1 Year After Coronary Stent Implantation. Circulation.

[9] Yang S, Kang J, Park KW, Hur SH, Lee NH, Hwang D (2023). Comparison of Antiplatelet Monotherapies After Percutaneous Coronary Intervention According to Clinical, Ischemic, and Bleeding Risks. J Am Coll Cardiol.

[10] Chen C, Fan H, Shuai R (2025). Analysis of risk factors for gastrointestinal bleeding in percutaneous coronary intervention patients treated with dual antiplatelet therapy after surgery. Medicine (Baltimore).

[11] Capodanno D, Angiolillo DJ (2023). Personalised antiplatelet therapies for coronary artery disease: what the future holds. Eur Heart J.

[12] Chandiramani R, Spirito A, Johnson JW, Mehta A, Vogel B, Faillace RT (2023). Antiplatelet therapy for coronary artery disease in 2023: current status and future prospects. Expert Rev Cardiovasc Ther.

[13] Pickett SJ, Levine GN, Jneid H, Bhatt DL, Nambi V Is there an optimal antiplatelet strategy after gastrointestinal bleeding in patients with coronary artery disease?Cardiology. 2021.

[14] Pal M, Parija S, Panda G, Dhama K, Mohapatra RK (2022). Risk prediction of cardiovascular disease using machine learning classifiers. Open Med Wars Pol.

[15] Liu T, Krentz A, Lu L, Curcin V (2025). Machine learning based prediction models for cardiovascular disease risk using electronic health records data: systematic review and meta-analysis. Eur Heart J Digit Health.

[16] Al Jowf GI, Kolhar M (2025). Key factors in predictive analysis of cardiovascular risks in public health. Sci Rep.

[17] Yoshida R, Ishii H, Morishima I, Tanaka A, Morita Y, Takagi K (2019). Performance of HAS-BLED, ORBIT, PRECISE-DAPT, and PARIS risk score for predicting long-term bleeding events in patients taking an oral anticoagulant undergoing percutaneous coronary intervention. J Cardiol.

[18] Costa F, van Klaveren D, James S, Heg D, Räber L, Feres F (2017). Derivation and validation of the predicting bleeding complications in patients undergoing stent implantation and subsequent dual antiplatelet therapy (PRECISE-DAPT) score: a pooled analysis of individual-patient datasets from clinical trials. Lancet.

[19] Tamis-Holland JE, Jneid H, Reynolds HR, Agewall S, Brilakis ES, Brown TM (2019). Contemporary Diagnosis and Management of Patients with Myocardial Infarction in the Absence of Obstructive Coronary Artery Disease: A Scientific Statement from the American Heart Association. Circulation.

[20] Mangieri A, Gallo F, Sticchi A, Khokhar AA, Laricchia A, Giannini F (2020). Dual antiplatelet therapy in coronary artery disease: from the past to the future prospective. Cardiovasc Interv Ther.

[21] Sabouret P, Savage MP, Fischman D, Costa F (2021). Complexity of Antiplatelet Therapy in Coronary Artery Disease Patients. Am J Cardiovasc Drugs Drugs Devices Interv.

[22] Samuelsen PJ, Eggen AE, Steigen T, Wilsgaard T, Kristensen A, Skogsholm A (2021). Incidence and risk factors for major bleeding among patients undergoing percutaneous coronary intervention: Findings from the Norwegian Coronary Stent Trial (NORSTENT). PloS One.

[23] Cheng Y, Liu X, Zhao Y, Sun Y, Zhang D, Liu F (2020). Risk Factors for Postoperative Events in Patients on Antiplatelet Therapy Undergoing Off-Pump Coronary Artery Bypass Grafting Surgery. Angiology.

[24] Rémond D, Shahar DR, Gille D, Pinto P, Kachal J, Peyron MA (2015). Understanding the gastrointestinal tract of the elderly to develop dietary solutions that prevent malnutrition. Oncotarget.

[25] Sorrentino S, Sartori S, Baber U, Claessen BE, Giustino G, Chandrasekhar J (2020). Bleeding risk, dual antiplatelet therapy cessation, and adverse events after percutaneous coronary intervention: The PARIS registry. Circ Cardiovasc Interv.

[26] Tsuji K, Uchida N, Nakanoh H, Fukushima K, Haraguchi S, Kitamura S (2024). The Gut-Kidney Axis in Chronic Kidney Diseases. Diagn Basel Switz.

[27] Modig S, Lannering C, Östgren CJ, Mölstad S, Midlöv P (2011). The assessment of renal function in relation to the use of drugs in elderly in nursing homes;a cohort study. BMC Geriatr.

[28] Dimitriadis K, Pyrpyris N, Iliakis P, Kanatas P, Theofilis P, Sakalidis A (2025). Optimal management of high bleeding risk patients undergoing percutaneous coronary interventions: Where do we stand?. J Cardiol.

[29] Zhang M, Liu D, Wang Q, Geng X, Hou Q, Gu G (2021). Gastrointestinal bleeding in patients admitted to cardiology: risk factors and a new risk score. Hell J Cardiol HJC Hell Kardiologike Epitheorese.

[30] Nopp S, Spielvogel CP, Schmaldienst S, Klauser-Braun R, Lorenz M, Bauer BN (2022). Bleeding risk assessment in end-stage kidney disease: Validation of existing risk scores and evaluation of a machine learning-based approach. Thromb Haemost.

[31] Lutz J, Menke J, Sollinger D, Schinzel H, Thürmel K (2014). Haemostasis in chronic kidney disease. Nephrol Dial Transplant.

[32] Jones A, Swan D, Lisman T, Barnes GD, Thachil J (2024). Anticoagulation in chronic kidney disease: Current status and future perspectives. J Thromb Haemost.

[33] Natale P, Palmer SC, Saglimbene VM, Ruospo M, Razavian M, Craig JC (2022). Antiplatelet agents for chronic kidney disease. Cochrane Database Syst Rev.

[34] Baaten CCFMJ, Schröer JR, Floege J, Marx N, Jankowski J, Berger M (2022). Platelet Abnormalities in CKD and Their Implications for Antiplatelet Therapy. Clin J Am Soc Nephrol.

[35] Molino D, De Lucia D, Gaspare De Santo N (2006). Coagulation disorders in uremia. Semin Nephrol.

[36] Nakamura M, Iijima R (2021). Implications and characteristics of high bleeding risk in East Asian patients undergoing percutaneous coronary intervention: Start with what is right rather than what is acceptable. J Cardiol.

[37] Vries MJA, Van der Meijden PEJ, Henskens YMC, Ten Cate-Hoek AJ, Ten Cate H (2016). Assessment of bleeding risk in patients with coronary artery disease on dual antiplatelet therapy. A systematic review. Thromb Haemost.

[38] Murali S, Vogrin S, Noaman S, Dinh DT, Brennan AL, Lefkovits J (2020). Bleeding severity in percutaneous coronary intervention (PCI) and its impact on short-term clinical outcomes. J Clin Med.

[39] Chowdhury MRK, Stub D, Dinh D, Karim MN, Chowdhury HA, Billah B (2025). Preoperative factors associated with in-hospital major bleeding after percutaneous coronary intervention: A systematic review. Heart Lung Circ.

[40] Yang X, Lu N, Yang L, Li B, Zhou W, Li Y (2025). Impact of prolonged cardiopulmonary bypass on gastrointestinal complications in cardiac surgery: a retrospective cohort study. Perioper Med.

[41] Wang L, Pei D, Ouyang YQ, Nie X (2019). Meta-analysis of risk and protective factors for gastrointestinal bleeding after percutaneous coronary intervention. Int J Nurs Pract.

[42] Collet JP, Thiele H, Barbato E, Barthélémy O, Bauersachs J, Bhatt DL (2021). 2020 ESC Guidelines for the management of acute coronary syndromes in patients presenting without persistent ST-segment elevation. Rev Esp Cardiol (Engl Ed).

[43] Saeed A, Haider M, Yousuf S, Ahmad S, Fine M, Yazdani A (2025). Role of proton pump inhibitors in prevention of upper gastrointestinal bleeding in patients on dual antiplatelet therapy: Systematic review and meta-analysis. Am J Ther.

[44] Yu C, Natarajan P, Patel AP, Bhatia HS, Khera AV, Neumann JT (2025). Polygenic risk, aspirin, and primary prevention of coronary artery disease. Eur Heart J Cardiovasc Pharmacother.

